# Deubiquitinating Enzyme OTUDs: Focus on Cancers and Antiviral Response

**DOI:** 10.32604/or.2025.063644

**Published:** 2025-09-26

**Authors:** Lang Chen, Rui Dong, Xuan Huan

**Affiliations:** 1 Department of General Surgery, Tangdu Hospital, Fourth Military Medical University, Xi’an, 710038, China; 2 Department of Geriatric Medicine, Tangdu Hospital, Fourth Military Medical University, Xi’an, 710038, China

**Keywords:** Deubiquitinating enzymes (DUBs), OTUDs, cancer, antiviral response

## Abstract

Deubiquitinating enzymes (DUBs) are key enzymes capable of cleaving ubiquitin chains and synergizing with ubiquitination modifications to regulate the function of key proteins and maintain normal physiological functions. OTUDs are a key subfamily of the ovarian tumor protease (OTU) family, with important DUB activities, and include mainly OTUD1, OTUD2, OTUD3, OTUD4, OTUD5, OTUD6A, and OTUD6B. In recent years, research on OTUD proteins has been gradually emphasized, and their aberrant expression has demonstrated significant research value in many diseases, such as cancer, immune abnormalities, neurological disorders, and embryonic developmental abnormalities. Therefore, a comprehensive understanding of the regulatory mechanisms of OTUD proteins and their use as therapeutic targets for diseases is of great value. This article focuses on the role of individual OTUD proteins in cancer progression and antiviral response. Importantly, in the context of cancer, we elaborate on their deubiquitinated protein targets and summarize the signaling mechanisms by which they promote or inhibit cancer progression. In the future, targeting OTUD proteins may become a therapeutic direction for cancer, and this review may be useful for research related to OTUD proteins and cancer. At present, there is a lack of research on targeted inhibitors or activators of OTUDs. More *in vivo* and *in vitro* studies on OTUDs may contribute to the development of inhibitors or agonists targeting OTUDs. Of course, when conducting these studies, researchers also need to pay attention to the impact of OTUDs on the host’s antiviral immune response.

## 1 Introduction to Ubiquitination

The ubiquitin-proteasome system (UPS) is a key pathway that regulates the degradation of proteins that undergo misfolding and oxidative damage in eukaryotic cells, including ubiquitination of proteins and degradation of proteasomes [1]. Ubiquitin (Ub) is a polypeptide with 76 amino acids highly conserved in eukaryotes. Ubiquitination of proteins is the process by which the C-terminal glycine residue of Ub is covalently linked to a lysine residue in the substrate protein [2]. This process belongs to an ATP-dependent post-translational modification, which plays a key role in regulating signaling, cell cycle, apoptosis, protein translocation, and DNA damage repair [3]. Ubiquitination is tightly controlled by the so-called three-step enzyme-catalyzed process, including ubiquitin-activating enzymes (E1), ubiquitin-coupling enzymes (E2), and ubiquitin ligases (E3), whose sequential modification of the substrate proteins determines the final function of the protein [4]. Driven by ATP, the cysteine residue on E1 forms a high-energy thioester bond with Ub; then E1 transfers Ub to the cysteine residue on E2; finally, E3 assists E2 in recognizing target proteins and transfers E2-bound Ub to the lysine residue of a specific target protein, and this is repeated until a complete Ub chain is formed [1,2].

In addition, ubiquitination modification not only regulates protein degradation; it is also more important in regulating protein translocation, activity, and function, which largely depends on the structure and length of the Ub chain. Due to the presence of seven lysine residues (Lys6, Lys11, Lys27, Lys29, Lys33, Lys48, and Lys63) and one methionine residue in the Ub, each of these residues can serve as an acceptor site for the next Ub [2–4]. Thus, in addition to mono-ubiquitination, substrate proteins can be linked to various poly Ub chains (linear and interlocking structures), leading to proteins exhibiting different biological outcomes and providing a structural basis for biological signal transduction. Topologies such as K6, K11, K27, K29, K33, K48, and K63 are common poly Ub chains that produce different endings for modifying substrate proteins [1]. For example, the K11-type poly Ub chain participates in the cell cycle and endoplasmic reticulum-associated degradation pathways; K27- and K29-type poly Ub chains are associated with lysosomal degradation pathways, contributing to cell survival [5,6]. The K48-type poly Ub chain is the most common type that activates proteasomal degradation signaling [7]. Furthermore, the K63-type poly Ub chain is involved in protein activation, translocation, and DNA damage repair processes [8]. Thus, ubiquitination modifications are involved in a wide range of biological functions, and dysregulation of the ubiquitination process may be involved in discovering a wide range of human diseases, especially cancer. In cancer, abnormal ubiquitination modifications form different Ub chains, which further promote the growth, metastasis, and immune evasion of cancer cells by regulating the expression of oncoproteins or tumor suppressor proteins.

## 2 Members of the OTUDs Subfamily of Deubiquitinating Enzymes

Protein ubiquitination is a highly conserved reversible post-translational modification process. The E1/E2/E3 axis attaches Ub chains to proteins, initiating the UPS. In contrast, DUBs can specifically remove Ub chains from proteins and block the UPS [8]. Thus, the E1/E2/E3 axis and DUBs jointly regulate the stability of protein function, and dysregulation of the expression of any of them may result in disease. With the continuous exploration of DUBs, humans have identified about 100 deubiquitinating enzymes, and the DUB members encoded by eukaryotic genes are mainly classified into seven families [8]. Among them, the ubiquitin-specific proteases, ubiquitin C-terminal hydrolases, OTUs, motif-interacting with ubiquitin-containing proteases, zinc-finger contain-ing ubiquitin peptidase 1 and Machado-Josephin domain proteases rely on their cysteine protease activity to cleave the Ub chain. In contrast, the JAMM/MPN domain-associated Zn-dependent metalloproteases family depends on their zinc metalloproteinase activity to cleave the Ub chain [4,8–10].

The OTUs family is the second largest superfamily of DUBs, containing 17 OTU members, and it is subdivided into four subfamilies: the otubains subfamily (OTUB1 and OTUB2), the OTUD subfamily (OTUD1, OTUD2, OTUD3, OTUD4, OTUD5, OTUD6A, OTUD6B, and ALG13), the A20-like OTUs subfamily (A20, OTUD7A, OTUD7B, TRABID, and VCPIP1), and the OTULIN subfamily (OTULIN and FAM105A) [10]. Characteristically, the human OTU family contains an OTU catalytic structural domain and other structural domains, such as ubiquitin-interacting motif (UIM), ubiquitin regulatory X (UBX), ubiquitin-associated (UBA), and zinc finger (ZNF) [10]. Among them, the OTUDs subfamily is a novel population of the OTU family, which possesses highly efficient deubiquitinating enzyme activities [10]. The OTU catalytic domains, which are the structural basis for their deubiquitinating effects, show more interest in the interlocking Ub chains [11]. Well, nowadays, cancer and immune diseases are still the most difficult diseases to overcome in the world, seriously reducing the quality of life and even the survival rate of patients. Research on OTUD proteins has gradually been emphasized in recent years, and significant progress has been made. Except for the ALG13 protein, we found that aberrant expression of seven other OTUD proteins is involved in multiple cancer progression and antiviral response processes. In this article, we summarize the roles and relationships of OTUD proteins in different cancers and antiviral responses, respectively, and elaborate on the deubiquitinated protein targets of OTUDs, focusing on the signaling mechanisms by which they promote or inhibit cancer progression.

## 3 New Frontiers of OTUD Proteins in Cancer Progression

### 3.1 OTUD1

OTUD1, or DUBA7, consists of 481 amino acids, including the OTU structural and C-terminal UIM structural domains [10]. It has been reported that the deletion of the UIM structural domain, which is indispensable for the cleavage of K63-type polyUb chains by OTUD1, severely affects the deubiquitination function of OTUD1 [11,12]. The research on OTUD1 and cancer has become increasingly close in recent years. In 2014, Carneiro et al. identified and characterized OTUD1 by single-chain variable fragment antibody-antigen assay and named it DUBA7, which is differentially expressed in thyroid cancer and can be used as a biomarker for early thyroid cancer, but the authors did not study the effect of OTUD1 on thyroid cancer progression in-depth [13]. Later, OTUD1 was identified as a novel stabilizer of the tumor suppressor protein P53. OTUD1 preferentially cleaves the K48-type polyUb chain of P53 protein and increases its stability, promoting apoptosis and inhibiting colony formation, suggesting that OTUD1 may inhibit cancer growth [14].

During cancer progression, transforming growth factor-β (TGF-β) sequentially activates SMAD2/3 and SMAD4 through activation of TGF-β type I receptor (TβRI), and SMAD4 enters the nucleus and promotes the expression of oncogenes, leading to epithelial-mesenchymal transition (EMT) [15]. At the same time, SMAD7 antagonizes TGF-β signaling by recruiting the E3 ubiquitin ligase Smurf2 to promote TβRI ubiquitination and degradation [16,17]. A study found that OTUD1 displays abnormally low expression in breast cancer, which is associated with cancer cell metastasis and lower survival [18]. OTUD1 first prevents the degradation of SMAD7 by cleaving its K48-type poly Ub chain and then promotes the formation of SMAD7/Smurf2 complex by cleaving its K33-type poly Ub chain, thus antagonizing the TGF-β/TβRI/SMAD signaling pathway, ultimately inhibiting EMT and cancer stem cell properties in breast cancer [18].

Apoptosis-inducing factor (AIF) is normally mainly confined to mitochondria and promotes cell survival by stabilizing the normal structure of mitochondria by maintaining the oxidative phosphorylation (OXPHOS) process [19,20]. However, when AIF translocates from mitochondria to the nucleus, it can activate the caspase-independent pro-apoptotic pathway to promote apoptosis by binding to and degrading chromosomal DNA, known as parthanatos [21,22]. Low OTUD1 expression has been reported to be associated with chemoresistance and poor prognosis in esophageal squamous cell carcinoma (ESCC) patients, while wild-type OTUD1 xenografts inhibit ESCC growth and promote chemosensitivity [22]. In mechanistic studies, it was confirmed that in mitochondria, OTUD1 can cleave the K27-type and K63-type poly-Ub chains of AIF on K244, which would lead to mitochondrial structural disruption and impairment of the OXPHOS process and ultimately induce ESCC cell death [22]. Alternatively, OTUD1 can indirectly promote the ubiquitination and degradation of the K48-type polyUb chain of the anti-apoptotic protein MCL1 by stabilizing DDB1 and CUL4-associated factor 10 (DCAF10), which increases the permeability of the mitochondrial membrane and promotes the release of cytochrome C and AIF, which is followed by the activation of the caspase-dependent apoptosis process through the cytochrome C [22]. In contrast, cleavage of AIF by OTUD1 at the K63-type polyUb chain on K255 encourages the entry of AIF into the nucleus and strongly binds chromosomal DNA, thereby inducing parthanatos [22]. This study illustrates that OTUD1 can promote apoptosis in cancer cells by activating caspase-independent and caspase-dependent apoptotic pathways.

In addition, OTUD1 was also significantly under-expressed in renal clear cell carcinoma (ccRCC) tissues, and low OTUD1 expression was associated with shorter disease-free survival and poor prognostic factors for overall survival (OS) in ccRCC patients [23,24]. Liu et al. demonstrated that OTUD1 overexpression significantly inhibited ccRCC cell proliferation and cell-cycle progression and that tumors of OTUD1-knockout nude mice grew more rapidly [23]. They further showed that OTUD1 prevented the degradation of PTEN protein and stabilized it by cleaving the K48-type poly Ub chain of PTEN protein. The stabilized PTEN inhibited the proliferation of ccRCC cells by suppressing the phosphatidylinositol 3 kinase/protein kinase B (PI3K/AKT) and tumor necrosis factor-α/nuclear factor kappa-B (TNF-α/NF-kB) signaling pathways, and at the same time, enhanced the sensitivity of cancer cells to tyrosine kinase inhibitors (TKIs) [23]. Later, it was demonstrated that OTUD1 could also induce apoptosis in ccRCC cells by deubiquitinating and stabilizing Bim proteins with pro-apoptotic effects [25].

Iron is an essential element for cell growth and proliferation, and while intracellular iron accumulation promotes cancer cell proliferation and metastasis, it also predisposes cancer cells to ferroptosis [26,27]. It has been demonstrated that excessive iron accumulation in cancer cells reacts with hydrogen peroxide and generates large amounts of reactive oxygen species (ROS), which are toxic to cancer cells and ultimately lead to the development of ferroptosis, which is one of the mechanisms that impede cancer progression [27,28]. Therefore, inducing ferroptosis in cancer cells is a novel cancer therapeutic target today [29]. Song et al. found that OTUD1 expression was significantly down-regulated in colorectal cancer and that OTUD1 deficiency blocked intracellular iron transport and suppressed tumor immunosurveillance, leading to an increased susceptibility to colorectal cancer [30]. It is suggested that the cancer-inhibitory effect of OTUD1 may be related to regulating the iron transport process. Transferrin receptor protein 1 (TFRC) is an important iron transporter protein, and iron response element binding protein 2 (IREB2) is involved in the regulation process of iron transporter proteins and activated IREB2 promotes cellular iron uptake by enhancing the expression of TFRC [31]. Studies in colorectal cancer confirmed that OTUD1 cleaves the K48-type and K63-type polyUb chains of IREB2 protein, and stabilized IREB2 protein increases intracellular iron concentration through activation of TFRC, and the accumulation of excess iron leads to the continuous production of ROS, which ultimately induces ferroptosis in cancer cells [30]. Meanwhile, the authors found that OTUD1 could enhance the host’s T cell-mediated antitumor immune response by promoting damage-associated molecular pattern (DAMP) release [30].

The accumulation of Yes-associated protein (YAP) in the nucleus has been associated with cancer, and OTUD1 has been identified as a DUB that cleaves the K63-type polyUb chain of YAP and as a negative regulator of the nuclear translocation and activity of YAP [32]. It suggests that OTUD1 exerts an oncogenic effect that may be related to the regulation of YAP signaling. Compared with normal samples, the expression level of OTUD1 was significantly downregulated in non-small cell lung cancer (NSCLC) tissues and cell lines, whereas OTUD1 overexpression significantly inhibited the proliferation, migration, and invasion of NSCLC cells, as well as improved OS in NSCLC patients, suggesting that OTUD1 is an oncogene in NSCLC [33–36]. Studies in NSCLC (PC-9 cells) and erlotinib-resistant NSCLC (PC-1/ER) cell lines demonstrated that OTUD1 inactivated the SOX9/SPP1 axis by blocking the nuclear translocation and activity of YAP1, which ultimately reversed erlotinib resistance in NSCLC cells [33]. Alternatively, OTUD1 may inhibit the proliferation, migration, and invasion of NSCLC cells by deubiquitinating and stabilizing the tumor suppressors KLF4 and FHL1, but the authors did not delve into the ubiquitination site of KLF4 [35,36].

**Figure 1:**
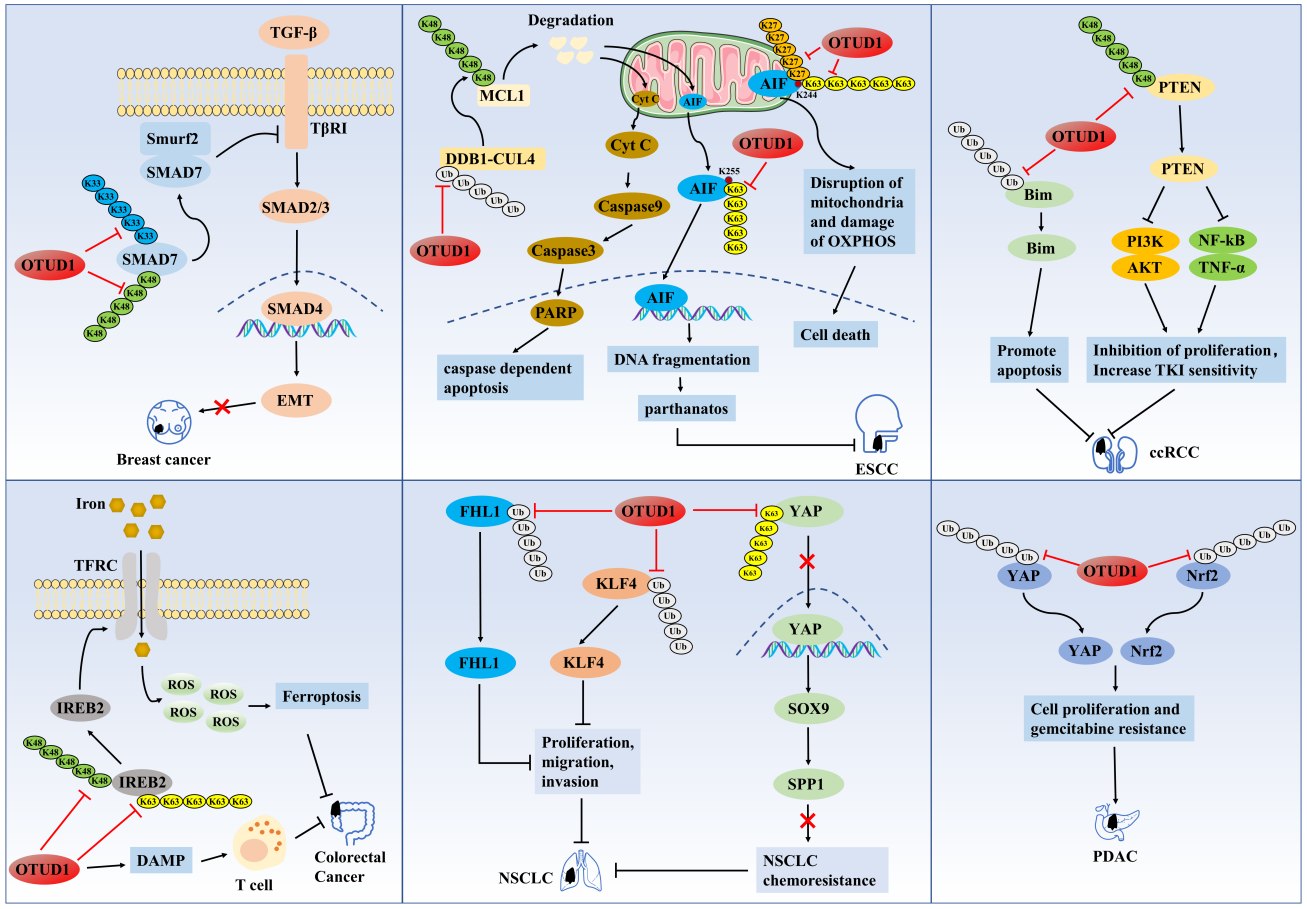
The role of OTUD1 in the pathogenesis of related cancers

However, it has also been suggested that OTUD1 may be an oncogene in some cancers. Analysis of the TCGA database showed that high OTUD1 expression was associated with worse overall survival in patients and may be an unfavorable prognostic factor for hepatocellular and ovarian cancers as well as specific subtypes of breast and cervical cancers [37]. We mentioned that OTUD1 can indirectly promote ubiquitination and degradation of the anti-apoptotic protein MCL1, which plays an oncogenic role in ESCC [22]. However, Wu demonstrated that OTUD1 can also directly interact with MCL1 protein to reverse the cell death process induced by sorafenib and BH3 mimetic inhibitors by deubiquitinating and stabilizing MCL1 protein [37]. In addition, OTUD1 expression was higher in chemoresistant pancreatic ductal adenocarcinoma (PDAC) cells and lower in chemosensitive cells, and silencing of OTUD1 increased the sensitivity of PDAC cells to gemcitabine [38]. It is suggested that OTUD1 may be associated with the development of chemoresistance in PDAC. Mechanistically, OTUD1 may induce PDAC resistance to gemcitabine and promote cell growth by deubiquitinating and stabilizing nuclear factor erythroid 2-related factor 2 (Nrf2) and YAP proteins. Suggesting that inhibition of OTUD1 may be a therapeutic target to improve the efficacy of PDAC chemotherapy [38] (Fig. 1). In summary, OTUD1 relies on its deubiquitination to regulate the expression of a variety of downstream proteins, including P53, SMAD7, YAP, KLF4, FHL1, AIF, DCAF10, IREB2, PTEN, Bim, thus regulating tumor cell signaling and exerting significant tumor suppressor effects. However, OTUD1 exhibited tumor-promoting effects in PDAC cells, which may result from tissue specificity, and more studies are still needed to confirm the link between OTUD1 and PDAC further.

OTUD1 first prevents the degradation of SMAD7 by cleaving its K48-type poly Ub chain and then promotes the formation of SMAD7/Smurf2 complex by cleaving its K33-type poly Ub chain, thus antagonizing the TGF-β/TβRI/SMAD signaling pathway, ultimately inhibiting EMT and cancer stem cell properties in breast cancer. OTUD1 cleaves the K27-type and K63-type poly-Ub chains of AIF on K244, which leads to mitochondrial structural disruption and impairment of the OXPHOS process and ultimately induce ESCC cell death. Alternatively, OTUD1 indirectly promotes the ubiquitination and degradation of the K48-type polyUb chain of the anti-apoptotic protein MCL1 by stabilizing DDB1 and DCAF10, which increases the permeability of the mitochondrial membrane and promotes the release of cytochrome C and AIF, which is followed by the activation of the caspase-dependent apoptosis process through the cytochrome C. In contrast, cleavage of AIF by OTUD1 at the K63-type polyUb chain on K255 encourages the entry of AIF into the nucleus and strongly binds chromosomal DNA, thereby inducing parthanatos. OTUD1 prevents the degradation of PTEN protein by cleaving the K48-type poly Ub chain of PTEN protein. The stabilized PTEN inhibited the proliferation of ccRCC cells by suppressing the PI3K/AKT and TNF-alpha/NF-kB signaling pathways, and at the same time, enhanced the sensitivity of cancer cells to tyrosine kinase inhibitors (TKIs). OTUD1 cleaves the K48-type and K63-type polyUb chains of IREB2 protein, and stabilized IREB2 protein increases intracellular iron concentration through activation of transferrin receptor (TFRC), and the accumulation of excess iron leads to the continuous production of ROS, which ultimately induces ferroptosis in cancer cells. OTUD1 enhances the host’s T cell-mediated antitumor immune response by promoting damage-associated molecular pattern (DAMP) release. OTUD1 inactivates the SOX9/SPP1 axis by blocking the nuclear translocation and activity of YAP1, which ultimately reverses erlotinib resistance in NSCLC cells. OTUD1 may inhibit the proliferation, migration, and invasion of NSCLC cells by deubiquitinating and stabilizing the tumor suppressors KLF4 and FHL1. OTUD1 may induce PDAC resistance to gemcitabine and promote cell growth by deubiquitinating and stabilizing Nrf2 and YAP proteins.

### 3.2 OTUD2

OTUD2, also known as YOD1 or DUBA8, consists of 348 amino acids, including the N-terminal UBX structural domain, the central OTU structural domain, and the C-terminal ZNF structural domain [10,39]. Several previous studies have shown that OTUD2 is a cofactor and deubiquitinating enzyme of p97 protein, and its binding to p97 protein can activate the endoplasmic reticulum-associated degradation pathway, thereby alleviating cytotoxic stress from protein misfolding [40]. Subsequent studies have also revealed that the expression level of OTUD2 is elevated under various stress conditions, which alleviates cytotoxicity by mitigating the accumulation of abnormal proteins and is protective against neurodegenerative lesions [41]. In addition, OTUD2 is also involved in the regulatory process of host inflammatory response [42].

In recent years, the role of OTUD2 in cancer has also been increasingly emphasized, and it may become a prognostic biomarker for cancer. OTUD2 has achieved some research results in NSCLC, pancreatic cancer, and their oncogene [34,43]. Bioinformatics analysis showed that high expression levels of OTUD2 mRNA were associated with poorer overall survival in NSCLC patients, suggesting that OTUD2 may be an oncogene in NSCLC [34]. In addition, the expression level of OTUD2 protein in pancreatic cancer tissues was significantly higher than that in normal tissues adjacent to cancer, and the overexpression of OTUD2 not only promotes the proliferation and metastasis of pancreatic cancer cells but also correlates with the immunosuppressive tumor microenvironment, which is an unfavorable prognostic indicator for overall survival and recurrence-free survival [43]. Similarly, several studies have shown that OTUD2 may be an oncogene in ovarian, osteosarcoma, and prostate cancers, negatively regulated by miR-4429, and miR-21-5p [44–46]. On the contrary, OTUD2 may be an oncogene in cervical cancer, negatively regulated by miR-373 [47]. However, these studies did not delve into the effects and mechanisms of OTUD2 on the progression of tumor tissues or cell lines and the correlation between OTUD2 and prognosis. The next study should combine bioinformatics, cellular experiments, and animal experiments to explore in detail the specific expression and value of OTUD2 in these cancers.

**Figure 2:**
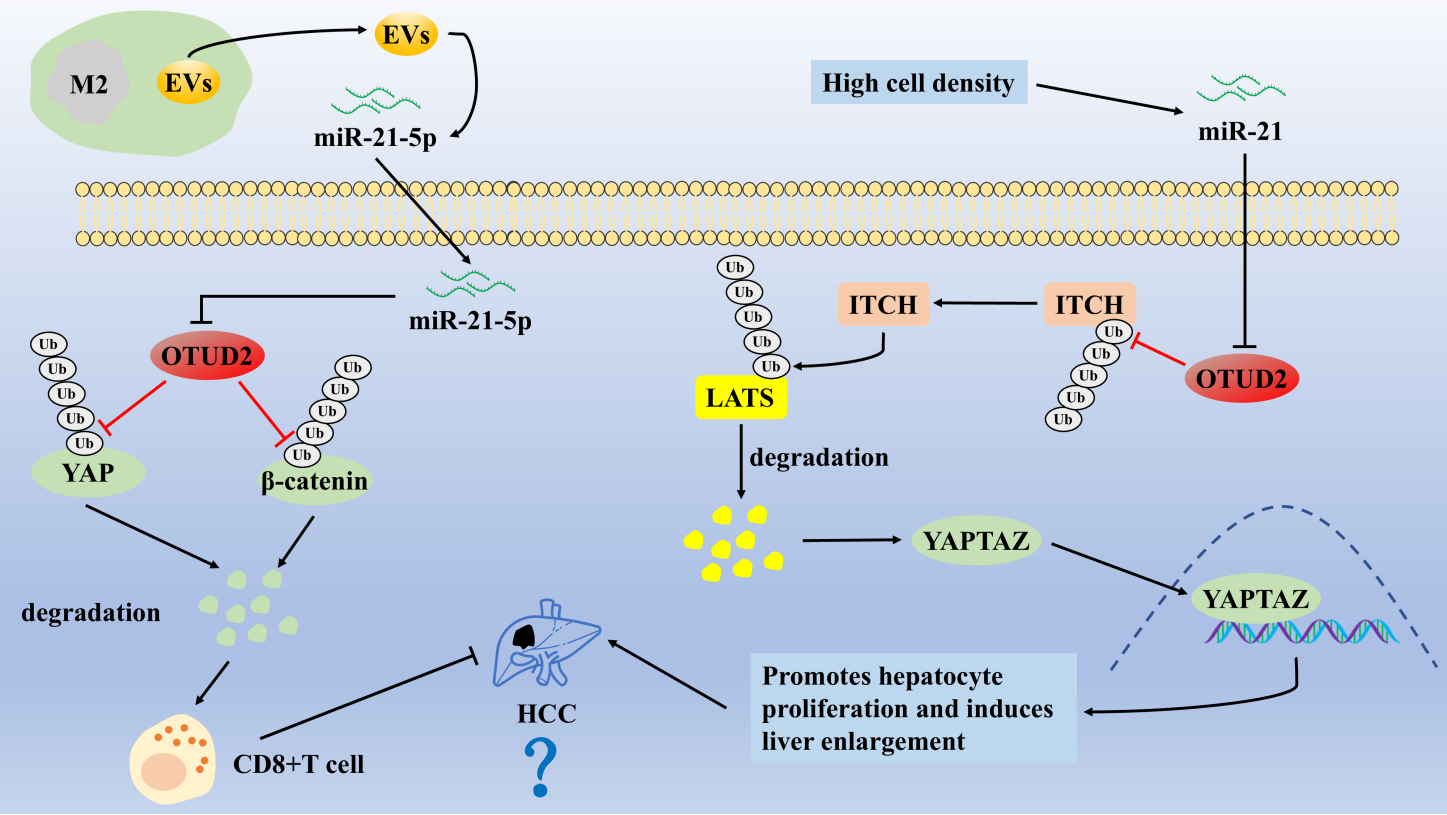
The role of OTUD2 in the pathogenesis of HCC

Currently, the link between OTUD2 and hepatocellular carcinoma (HCC) progression seems to be debated. Kim et al.’s research team found that the expression level of OTUD2 in hepatocellular carcinoma tissues was significantly higher than that in adjacent normal tissues and was strongly and positively correlated with the expression level of YAP [48]. They demonstrated that OTUD2 could promote LATS1/2 ubiquitination and degradation by enhancing the stability of ITCH (an E3 ligase), which induced the entry of YAP/TAZ into the nucleus, enhanced hepatocyte proliferation and induced hepatomegaly [48,49]. They suggested that OTUD2 is a positive regulator of the YAP/β-catenin pathway and may be an oncogene in HCC [48]. In contrast, a recent study demonstrated that M2-type macrophage-derived extracellular vesicles (EVs) target and inhibit OTUD2 by secreting miR-21-5p, which leads to activation of the YAP/β-catenin pathway and subsequent CD8^+^ T cell depletion, whereas OTUD2 overexpression enhances the CD8^+^ T-cells by inhibiting the YAP/β-catenin pathway’s antitumor effects, thereby inhibiting HCC [50] (Fig. 2). Thus, OTUD2 may, in turn, be a negative regulator of the YAP/β-catenin pathway, acting as an oncogene in HCC. However, these two studies may have certain limitations. We suggest that the relationship between OTUD2 and HCC can be jointly explored from the combined perspectives of cell lines, mouse cancer models, and clinical patients, such as complementing the effects of OTUD2 overexpression or knockdown on the behavior of HCC cells and the size of the lumps in mouse HCC models, as well as exploring the impact of OTUD2 on the YAP/β-catenin pathway. The regulatory mechanism of OTUD2 is also particularly necessary to analyze the correlation between OTUD2 expression in HCC tissues and prognosis in conjunction with bioinformatics, tissue and clinicopathological features of clinical HCC patients.

OTUD2 promotes LATS1/2 ubiquitination and degradation by enhancing the stability of ITCH (an E3 ligase), which induces the entry of YAP/TAZ into the nucleus, enhances hepatocyte proliferation, and induces hepatomegaly. M2-type macrophage-derived EVs target and inhibit OTUD2 by secreting miR-21-5p, which leads to activation of the YAP/β-catenin pathway and subsequent CD8^+^ T cell depletion, whereas OTUD2 overexpression enhances the CD8^+^ T-cells by inhibiting the YAP/β-catenin pathway’s antitumor effects, thereby inhibiting HCC.

### 3.3 OTUD3

OTUD3 is an acetylation-dependent deubiquitinating enzyme consisting of 398 amino acids, mainly including the OTU and UBA structural domains, which are highly conserved in eukaryotic evolution [10,51]. The deubiquitinating enzyme activity of OTUD3 has been reported to be tightly regulated by acetylation modifications, and acetylated OTUD3 is involved in physiological or pathological disease processes by removing the Ub chain of a variety of proteins, including cancers, antiviral responses, neurological disorders, embryonic development, and metabolic diseases [52].

**Figure 3:**
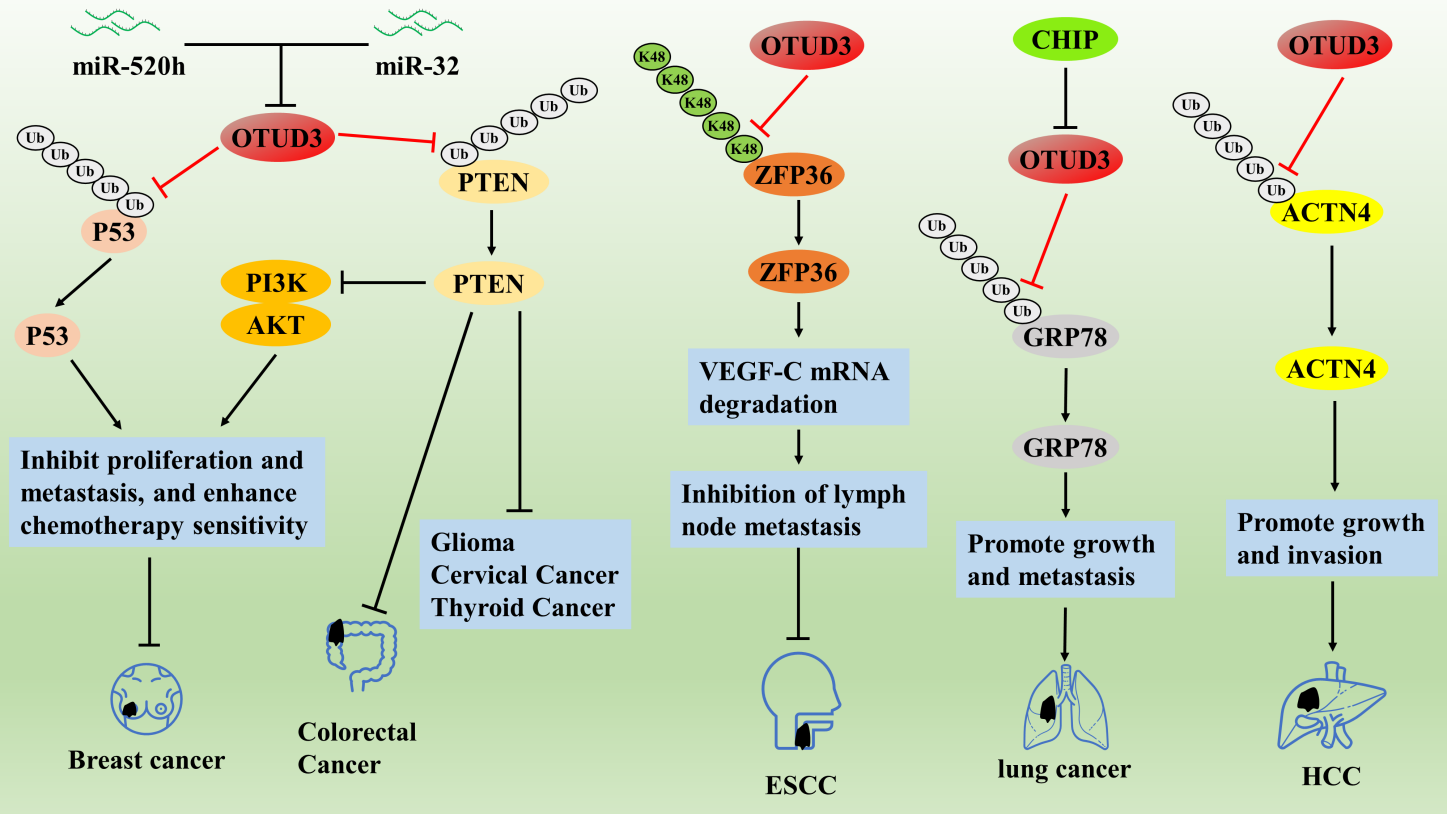
The role of OTUD3 in the pathogenesis of related cancers

Relevant evidence suggests that OTUD3 is strongly associated with the progression of several human cancers. Studies have confirmed that the OTUD3/PTEN axis inhibits the proliferation and metastasis of breast cancer cells by inhibiting the transduction of the PI3K/AKT pathway [53]. Whereas miR-520h induced paclitaxel resistance in breast cancer MCF-7 cells by inhibiting the OTUD3/PTEN axis, co-treatment with miR-520h inhibitor and OTUD3 overexpression significantly enhanced the sensitivity of MCF-7 cells to paclitaxel [54]. Similarly, the OTUD3/P53 axis inhibited breast cancer cell proliferation and enhanced chemosensitivity [55]. However, OTUD3 tends to show low expression in breast cancer tissues or cells and may be a novel cancer biomarker for metastasis and poor prognosis in breast cancer patients [55]. Studies have also reported that OTUD3 is lowly expressed in glioma, colorectal, cervical, and thyroid cancers, correlating with their poor prognosis, and OTUD3 also appears to inhibit cancer cell proliferation and metastasis by stabilizing PTEN proteins [56–58]. In conclusion, OTUD3 acts as a tumor suppressor in certain cancers, mainly in a PTEN and p53 protein-dependent manner. In addition, it has been demonstrated in colorectal cancer studies that the oncogene miR-32 can bind to the 3^′^-untranslated region of the OTU structural domain of OTUD3, directly targeting and inhibiting the transcription of OTUD3, thereby promoting the proliferation and invasion of colorectal cancer cells [59]. ZFP36 ring finger protein is an RNA binding protein that can inhibit cancer development by promoting mRNA degradation [60]. In studies on human esophageal cancer, OTUD3 directly interacted with ZFP36 and stabilized the ZFP36 protein by cleaving the K48-type polyUb chain, which subsequently promoted the degradation of vascular endothelial growth factor-C (VEGF-C) mRNA to inhibit lymphatic metastasis [61]. OTUD3 was significantly down-regulated in heavy smokers compared to non-smoking or normal tissues, and low OTUD3 expression was significantly associated with adverse clinicopathological features, including tumor size, lymph node metastasis, pathological grading, and clinical staging [61]. This is because nicotine blocks the OTUD3/ZFP36/VEGF-C axis and promotes lymphatic metastasis by downregulating OTUD3 expression [61] (Fig. 3). Therefore, smoking cessation may help improve the prognosis of esophageal cancer patients.

On the contrary, a related study reported that OTUD3 may act as an oncogene in HCC and lung cancer. OTUD3 was significantly overexpressed in HCC tissues compared with paracancerous tissues and was associated with larger tumor volume, more vascular infiltration, intrahepatic metastasis, worse TNM stage, and lower overall survival [62]. Both *in vivo* and *in vitro* experiments confirmed that OTUD3 promoted HCC growth and invasion through deubiquitination and stabilization of alpha actinin 4 (ACTN4) protein, whereas OTUD3 knockdown significantly inhibited the malignant behavior of HCC cells [62]. In addition, OTUD3 is also overexpressed in human lung cancer and is associated with poor prognosis in lung cancer patients; OTUD3 deficiency inhibits the oncogenic ability of lung cancer cells, while OTUD3 overexpression promotes the malignant progression of lung cancer [57,63]. Mechanistically, OTUD3 could not maintain the stability of PTEN proteins, but OTUD3 could promote lung cancer growth and metastasis by deubiquitinating and stabilizing the glucose regulatory protein GRP78 [57]. In addition, CHIP was identified as the E3 ubiquitin ligase of OTUD3, which can block OTUD3/GRP78 by accelerating the degradation of OTUD3 through ubiquitination, thus inhibiting the malignant progression of lung cancer [63] (Fig. 3). The above studies illustrate that OTUD3 inhibits the malignant progression of breast, glioma, colorectal, cervical, thyroid, and esophageal cancers; conversely, OTUD3 promotes the malignant progression of HCC and lung cancer.

OTUD3/PTEN axis inhibits the proliferation and metastasis of breast cancer cells by inhibiting the transduction of the PI3K/AKT pathway. The OTUD3/P53 axis inhibited breast cancer cell proliferation and enhanced chemosensitivity. OTUD3 directly interacts with ZFP36 and stabilizes the ZFP36 protein by cleaving the K48-type polyUb chain, which subsequently promotes the degradation of VEGF-C mRNA to inhibit lymphatic metastasis. OTUD3 promotes lung cancer growth and metastasis by deubiquitinating and stabilizing the glucose regulatory protein GRP78. OTUD3 promotes HCC growth and invasion through deubiquitination and stabilization of ACTN4 protein.

### 3.4 OTUD4

OTUD4 is a deubiquitinating enzyme with high sensitivity to K48-type polyUb chains, consisting of 1113 amino acids, and is involved in various physiological and pathological processes [10]. It has certain structural peculiarities in that, in addition to the OTU structural domain, it contains a putative Tudor structural domain, which is the structural basis for the interaction of OTUD4 with RNA or methylated histones [10]. In addition, OTUD4 is involved in the repair of DNA alkylation damage, and OTUD4 deletion increased the sensitivity of tumor cells to alkylating agents, suggesting that OTUD4 may be a novel target for enhancing the sensitivity of tumor cells to alkylating drugs [64].

**Figure 4:**
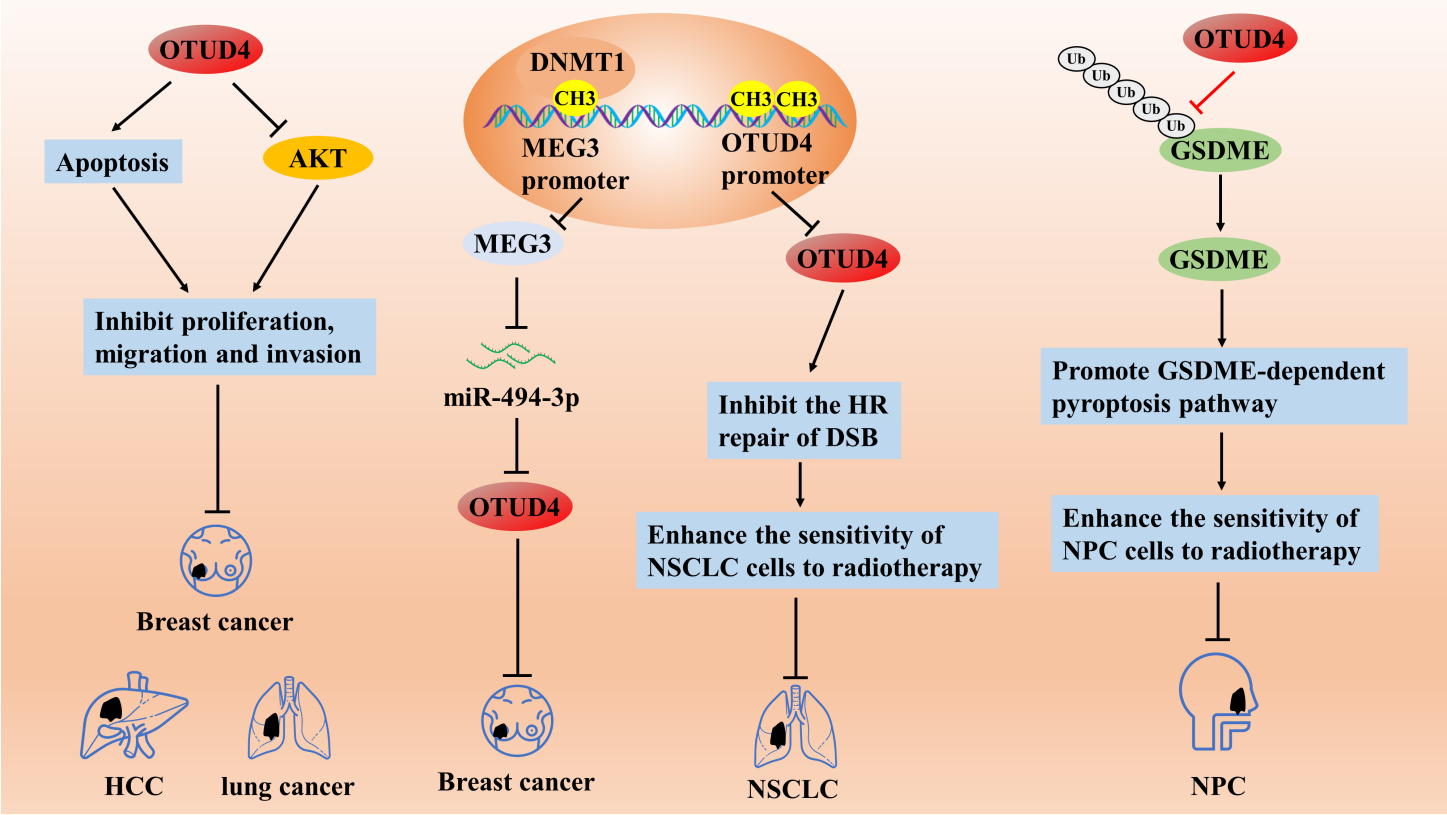
The role of OTUD4 in the pathogenesis of related cancers

Studies have shown that as a tumor suppressor, OTUD4 expression is significantly down-regulated in various solid tumors, and low OTUD4 expression is a predictive molecule for poor prognosis [65]. In cellular experiments concerning breast, liver, and lung cancers, it was found that OTUD4 overexpression may inhibit the proliferation, migration, and invasive ability of breast, liver, and lung cancer cells by promoting apoptosis and inhibiting the AKT signaling pathway in cancer cells [65]. In addition, OTUD4 expression may be regulated by methylation. In breast cancer, Zhu et al. demonstrated that the DNA methyltransferase DNMT1 induces hypermethylation of the promoter of maternally expressed gene 33 (MEG3) and inhibits its expression, which then promotes the growth of breast cancer cells through the regulation of the miR-494-3p/OTUD4 axis [66]. More directly, the expression level of OTUD4 was significantly reduced in NSCLC cell lines and tissues due to the inhibitory effect of hypermethylation of the promoter region on OTUD4, and low OTUD4 expression was significantly associated with poorer overall survival, first-progression survival, and post-progressionsurvival [67]. Mechanistically, OTUD4 could enhance the sensitivity of NSCLC cells to radiotherapy by inhibiting the homologous recombination repair pathway of DNA double-strand breaks (DSBs), but the specific regulatory mechanism was unclear [67]. Therefore, regulating the degree of methylation of the OTUD4 promoter region may be a therapeutic target for NSCLC. In addition, OTUD4 is down-regulated in radiotherapy-resistant nasopharyngeal carcinoma (NPC), leading to radiotherapy resistance and poor clinical outcomes; OTUD4 was identified as a deubiquitinating enzyme of gasdermin-E (GSDME) proteins, and overexpression of OTUD4 enhances radiosensitization of NPC cells by initiating a GSDME-dependent pyroptosis pathway [68] (Fig. 4). Thus, OTUD4 may be a potential therapeutic target for enhancing NPC radiosensitization. Differently, OTUD4 expression may be significantly upregulated in pancreatic ductal adenocarcinoma, and the overall survival time of low OTUD4-expressing patients was considerably longer than that of high-expressing patients, suggesting that OTUD4 may be a pro-carcinogenic gene in pancreatic ductal adenocarcinoma [64]. However, the specific role of OTUD4 in PDAC lacks confirmation from cellular and animal experiments, and further studies are needed.

OTUD4 inhibits the proliferation, migration, and invasive ability of breast, liver, and lung cancer cells by promoting apoptosis and inhibiting the AKT signaling pathway. The DNA methyltransferase DNMT1 induces hypermethylation of the promoter of MEG3 and inhibits its expression, which then promotes the growth of breast cancer cells through the regulation of the miR-494-3p/OTUD4 axis. OTUD4 can enhance the sensitivity of NSCLC cells to radiotherapy by inhibiting the homologous recombination (HR) repair pathway of DNA DSBs. OTUD4 enhances the radiosensitization of NPC cells by initiating a GSDME-dependent pyroptosis pathway.

### 3.5 OTUD5

OTUD5, also known as DUBA, consists of 571 amino acids and is about 60 kD, mainly consisting of the OTU structural and UIM structural domains at the C-terminus [10,69]. OTUD5, a phosphorylation-activation-dependent DUB, is involved in various processes, such as DNA damage repair, inflammatory response, and innate immune response, and it has also been achieved in cancer research [69,70].

Knockdown of OTUD5 has been reported to significantly promote the proliferation of HCC cells (Huh7 and Hep3B), and reduced levels of OTUD5 are associated with a highly invasive phenotype and poor clinical outcome and may play an oncogene role in a variety of solid tumors [71]. OTUD5 expression was significantly downregulated in HCC and NSCLC tissues compared to normal tissues, and low OTUD5 expression was associated with higher tumor grade, tumor size, and TNM stage [71]. Promyelocytic leukemia (PML) protein acts as a tumor suppressor by regulating P53 transcription, and they demonstrated that OTUD5 acts as a tumor suppressor by cleaving the K63-type polyUb chain inhibits TRIM25 activity, thereby suppressing TRIM25 enrichment on the PML promoter, which in turn promotes PML transcription and inhibits HCC and NSCLC progression [71]. In the face of genotoxic stress-induced DNA damage response, programmed cell death 5 (PDCD5) exerts significant anticancer effects by binding to P53 protein to induce apoptosis and maintain genome stability [72,73]. OTUD5 has been reported to be able to rely on its own UIM structural domain to interact with PDCD5 and P53 proteins and sequentially activate the PDCD5/p53 axis through deubiquitination in response to DNA damage stress [72,73].

In contrast, the knockdown of OTUD5 resulted in the loss of activity of PDCD5 and p53 proteins, promoting proliferation, metastasis, drug resistance, and apoptosis resistance in NSCLC cells [74]. In addition, overexpression of OTUD5 can also inhibit the proliferation, invasion, and migration of NSCLC cells by deubiquitinating and stabilizing PTEN proteins, while miR-652-3p acts as an upstream molecule targeting to inhibit OTUD5 expression [75]. In cervical cancer, low expression of OTUD5 was positively correlated with tumor stage, lymph node metastasis, and poor prognosis [76]. Cervical cancer C33A cells in the OTUD5 overexpression group were more sensitive to radiotherapy compared with the OTUD5-silenced group and the control group because OTUD5 overexpression reduces the level of AKT phosphorylation activation through deubiquitination to increase the sensitivity of cervical cancer to radiotherapy [77].

**Figure 5:**
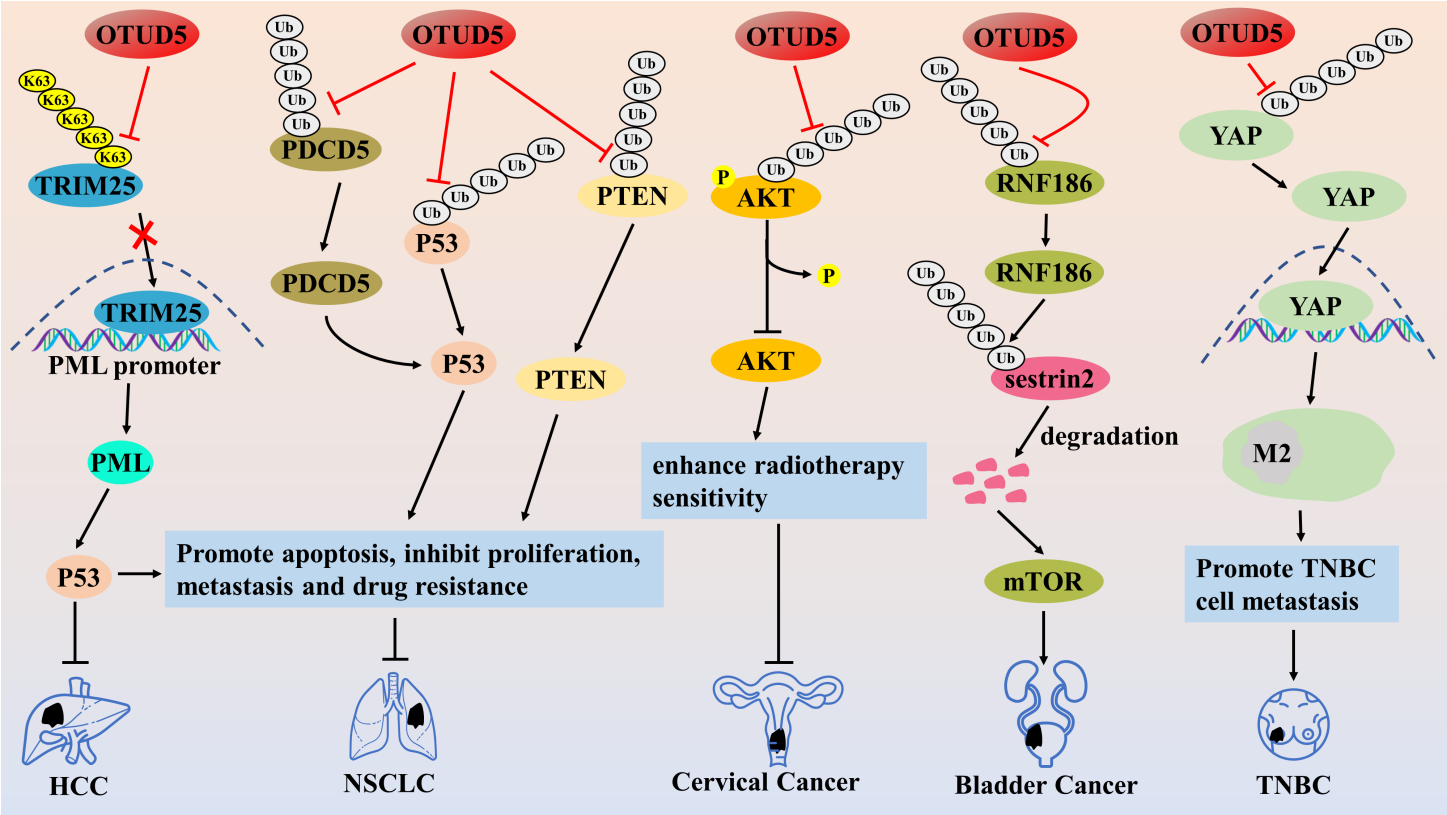
The role of OTUD5 in the pathogenesis of related cancers

However, some related studies have reported that OTUD5 acts as an oncogene in bladder cancer and triple-negative breast cancer (TNBC). Hou et al. found that OTUD5 was overexpressed in bladder cancer tissues or cell lines and that OTUD5 knockdown or overexpression inhibited and promoted cell proliferation in bladder cancer, respectively [78]. OTUD5 deubiquitinates and stabilizes the E3 ligase RNF186, degrading the sestrin2 protein by ubiquitination. Sestrin2 protein is an inhibitor of the mammalian target of rapamycin (mTOR) pathway, which leads to the activation of the mTOR signaling pathway, suggesting that OTUD5 is activated by regulating the OTUD5/RNF186/sestrin2/mTOR axis to promote bladder cancer progression [78]. In addition, OTUD5 knockdown also enhances the sensitivity of bladder cancer cells to everolimus, an inhibitor of mTOR [78]. This study provides new ideas for personalized treatment of bladder cancer patients, but the lack of specific inhibitors for OTUD5 does not allow an in-depth analysis of the therapeutic effects of OTUD5 inhibitors combined with everolimus. In addition, in TNBC cells, OTUD5 is a deubiquitinating enzyme of YAP protein, and TNBC cell-derived OTUD5 can significantly enhance the metastatic potential of TNBC by inducing M2-type macrophage polarization by promoting the nuclear translocation activity of YAP protein [79] (Fig. 5). Therefore, the authors concluded that OTUD5 acts as an oncogene in TNBC. However, this study still needs to be supplemented with certain experiments to draw such conclusions, including the expression of OTUD5 in TNBC cell lines and tissues, the effects of OTUD5 knockdown or overexpression on the malignant behavior of TNBC cells and tumor progression in mice, and the association between OTUD5 and clinical prognosis.

OTUD5 inhibits TRIM25 activity by cleaving the K63-type polyUb chain, thereby suppressing TRIM25 enrichment on the PML promoter, which in turn promotes PML transcription and inhibits HCC and NSCLC progression. OTUD5 inhibits the proliferation, invasion, and migration of NSCLC cells by deubiquitinating and stabilizing PDCD5/p53 axis and PTEN proteins. OTUD5 reduces the level of AKT phosphorylation activation through deubiquitination to increase the sensitivity of cervical cancer to radiotherapy. OTUD5 promotes bladder cancer progression by adjusting the RNF186/sestrin2/mTOR pathway. OTUD5 enhances the metastatic potential of TNBC by inducing M2-type macrophage polarization by promoting the nuclear translocation activity of YAP protein.

### 3.6 OTUD6A

**Figure 6:**
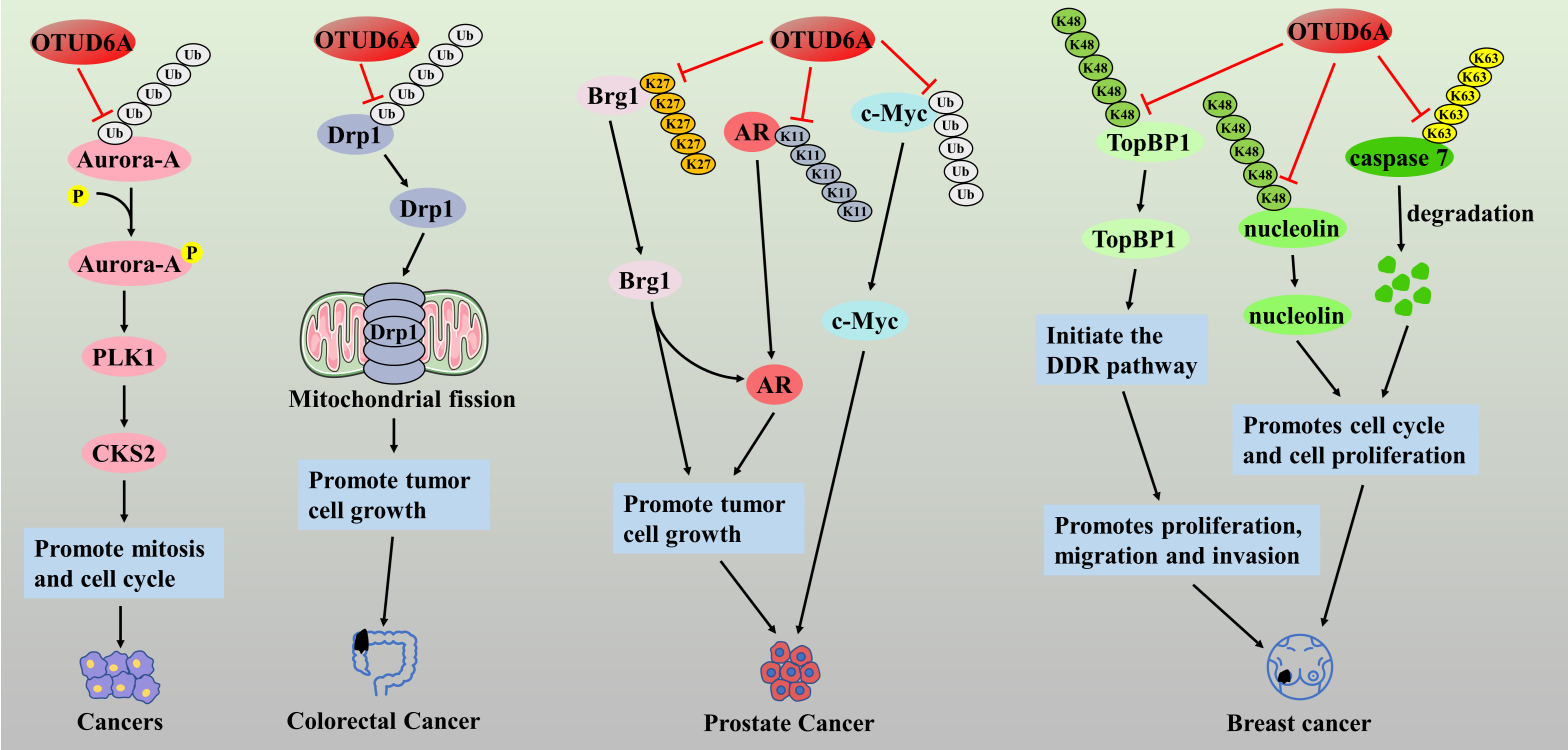
The role of OTUD6A in the pathogenesis of related cancers

In recent years, several studies have confirmed the involvement of OTUD6A in the malignant progression of colorectal, prostate, breast, and gastric cancers as an oncogene. Aurora kinase A (Aurora-A) is a serine/threonine kinase that is overexpressed in various human cancers, and inhibitors targeting Aurora kinase are under clinical investigation [80]. Kim et al. found that OTUD6A could enhance the stabilization and kinase activity of Aurora-A protein through deubiquitination, thereby promoting the development of Polo-like kinase 1 (PLK1) and cell cycle protein-dependent kinase. PLK1 and Cyclin-dependent kinases regulatory subunit 2 (CKS2) can drive mitosis and cell cycle progression in tumor cells through deubiquitination [81], suggesting that OTUD6A is a potential therapeutic target for cancer. Dynamin-related protein 1 (Drp1) is a mitochondrial fission protein associated with tumor cell growth, migration, and invasion [82]. It has been reported that the expression level of OTUD6A in colorectal cancer tissues is significantly higher than that in normal tissues, and OTUD6A can increase the stability and promote the expression of Drp1 protein through ubiquitination, which promotes the onset of Drp1-mediated mitochondrial fission, and enhances the proliferation and colony formation of cancer cells [83]. In addition, immunohistochemical analysis showed that OTUD6A protein was also specifically highly expressed in prostate cancer tissues, which was closely associated with higher prostate specific antigen (PSA), Gleason scores, and tumor stage in patients, as well as being a predictor of higher risk of postoperative recurrence and poorer survival [84,85]. The authors further found that OTUD6A could cleave the K27-type poly-Ub chain of the Brg1 protein and the K11-type poly-Ub chain of the androgen receptor (AR), preventing their degradation [84]. Both the Brg1 and AR proteins are pro-oncogenes in prostate cancer, and thus, OTUD6A could promote the proliferation of prostate cancer cells by deubiquitinating and stabilizing the brahma-related gene 1 (Brg1) and androgen receptor (AR) proteins. Similarly, OTUD6A may be a physiological DUB of the c-Myc oncoprotein, which promotes prostate carcinogenesis mainly by deubiquitinating and stabilizing the c-Myc protein [85]. In breast cancer, OTUD6A was found to accumulate in large numbers at DNA damage sites and increase the stability of DNA topoisomerase II-binding protein 1(TopBP1) by cleaving the K48-type polyUb chain, thereby initiating the DNA damage response (DDR) pathway, leading to tumor cell proliferation, migration, and invasion [86]. Similarly, overexpression of OTUD6A cleaved the K48-type polyUb chain of nucleolin, an oncogenic protein. It promoted its stabilization, while OTUD6A also cleaved the K63-type polyUb chain of caspase 7, an apoptotic caspase, and promoted its degradation, which enhanced the cell cycle progression and induced cell proliferation in breast cancer MCF7 cells [87] (Fig. 6). These studies suggest that OTUD6A is a tumor-promoting factor, and its tumor-suppressor effect has not yet been found OTUD6A may become an important cancer therapeutic target, and the study of inhibitors targeting OTUD6A may be a meaningful research direction.

OTUD6A drives mitosis and cell cycle by activating Aurora-A/PLK1/CKS2 signaling axis. OTUD6A can increase the stability and promote the expression of Drp1 protein through ubiquitination, which promotes the onset of Drp1-mediated mitochondrial fission, and enhances the proliferation and colony formation of cancer cells. OTUD6A promotes the proliferation of prostate cancer cells by deubiquitinating and stabilizing the Brg1 and AR proteins. OTUD6A promotes prostate carcinogenesis by deubiquitinating and stabilizing the c-Myc protein. In breast cancer, OTUD6A increases the stability of TopBP1 by cleaving the K48-type polyUb chain, thereby initiating the DDR pathway, leading to tumor cell proliferation, migration, and invasion. OTUD6A cleaves the K48-type polyUb chain of nucleolin and the K63-type polyUb chain of caspase 7, which enhanced the cell cycle progression and induced cell proliferation in breast cancer MCF7 cells.

### 3.7 OTUD6B

*In vitro* cellular experiments confirmed that the expression level of OTUD6B was significantly higher in lung cancer than in normal tissues, and OTUD6B knockdown inhibited the proliferation, migration, and invasion of lung cancer cells (A549 and Pc9 cells) [88]. Interestingly, OTUD6B-1 and OTUD6B-2, the two splice isoforms of OTUD6B, regulated protein synthesis in diametrically opposite ways, with OTUD6B-1 inhibiting protein synthesis and OTUD6B-2 promoting protein synthesis [89]. The OTUD6B-1/OTUD6B-2 mRNA ratio was reduced in NSCLC tissues compared to normal tissues, and OTUD6B-2 may promote the growth and proliferation of NSCLC cells by promoting the expression of cell cycle protein D1, which is a novel therapeutic target and a potential biomarker of NSCLC [89]. Given that inhibition of protein synthesis is a viable therapeutic strategy for NSCLC, modulation of the transformation of OTUD6B isoforms may be a novel therapeutic target. Recent pan-cancer analyses have also confirmed that OTUD6B is expressed at relatively higher levels in most tumors than in normal tissues and that high OTUD6B expression is significantly associated with a poor prognosis in patients with lung adenocarcinoma, breast invasive carcinoma, hepatocellular carcinoma, and thyroid cancer [88]. Therefore, the authors concluded that OTUD6B acts as an oncogene in most tumors.

**Figure 7:**
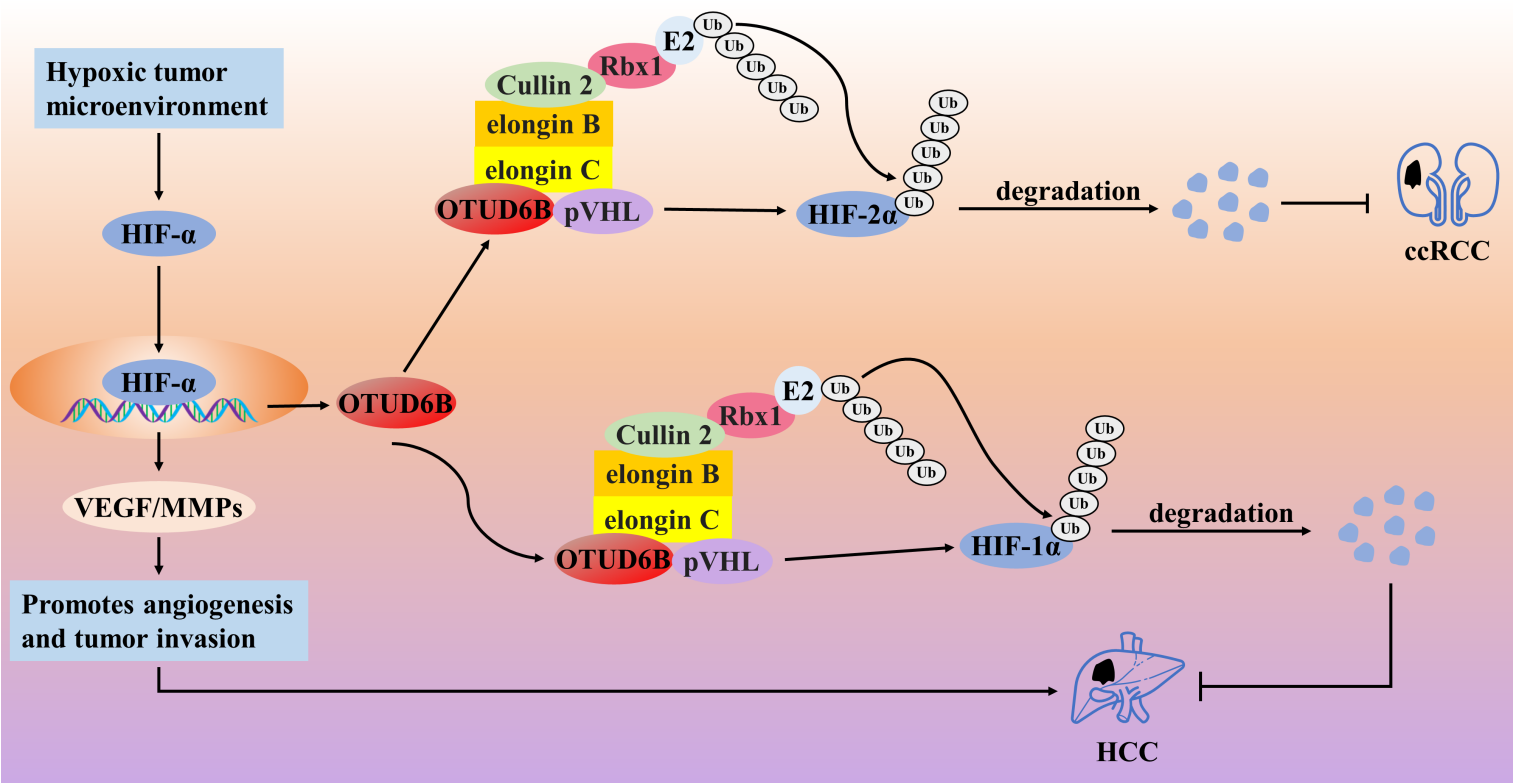
The role of OTUD6B in the pathogenesis of related cancers

In contrast, OTUD6B may be a tumor suppressor factor in HCC and ccRCC. The von Hippel–Lindau (VHL) tumor suppressor (pVHL) protein is a tumor suppressor that promotes the degradation of hypoxia-inducible factor-α (HIF-α) [90,91]. The Cul2-elonginB/C (CBC) complex consists of Cullin 2, elongin B, elongin C, and Rbx1 [92]. It has been shown that pVHL binds to elonginB/C and forms the VHL-CBC ligase complex, which protects pVHL from proteasomal degradation [90]. However, mutation of pVHL severely destabilizes the binding of pVHL to elonginB/C, leading to ubiquitination and degradation of pVHL; when pVHL is degraded or absent, HIF-α accumulates in the nucleus and subsequently promotes various tumors by activating the transcription of various oncogenic molecules, which is manifested as the VHL syndrome [92–94]. Therefore, maintaining the stability of pVHL is a therapeutic target for blocking the HIF-α signaling axis. In HCC cells, the knockdown of OTUD6B promoted the migratory ability and EMT of HCC cells but did not affect the proliferative ability of the cells; in the HCC nude mouse model, knockdown of OTUD6B also promoted angiogenesis and lung metastasis but did not affect tumor growth [95]. Immunohistochemical analysis also showed that the expression level of OTUD6B protein gradually decreased with increasing pathological stage of HCC, and low expression of OTUD6B was positively correlated with shorter overall survival and disease-free survival [95]. This suggests that OTUD6B acts as a tumor suppressor factor in HCC. Mechanistically, in the hypoxic tumor microenvironment, HIF-1α promotes angiogenesis and tumor invasion and directly promotes OTUD6B transcription. In turn, upregulated OTUD6B was able to prevent the degradation of pVHL protein by maintaining the stability of the pVHL-CBC ligase complex, which in turn inhibited the activation of the HIF-1α signaling axis and ultimately inhibited the migration and metastasis of HCC cells, suggesting the existence of negative feedback regulation between HIF-1α/OTUD6B/pVHL [95,96]. Interestingly, the stabilization of pVHL protein by OTUD6B was not dependent on its deubiquitinating enzyme activity. Similarly, in ccRCC tumor cells with pVHL missense mutations, OTUD6B inhibited ccRCC migration by regulating pVHL/HIF-2α; low levels of OTUD6B predicted a higher pathological stage and shorter OS in ccRCC patients [94] (Fig. 7). Considering that the two shear isoforms of OTUD6B have opposing roles in cancer progression, this may explain the opposite roles of OTUD6B in different cancer types. Investigating the roles of OTUD6B-1 and OTUD6B-2 in cancer separately might more accurately assess the link between OTUD6B and cancer progression, thus opening up a new field of cancer-specific therapy.

In the hypoxic tumor microenvironment, HIF-1α promotes angiogenesis and tumor invasion and directly promotes OTUD6B transcription. In turn, OTUD6B prevents the degradation of pVHL protein by maintaining the stability of the pVHL-CBC ligase complex, which in turn inhibits the activation of the HIF-1α signaling axis and ultimately inhibits the migration and metastasis of HCC cells. OTUD6B inhibited ccRCC migration by regulating pVHL/HIF-2α.

## 4 Role of OTUD Proteins in the Antiviral Response

In the face of viral infection, an inadequate antiviral response can lead to chronic illness, while an overactive response can lead to the development of inflammatory and even autoimmune diseases. Therefore, the host must have mechanisms to regulate the antiviral response to maintain immune homeostasis tightly. Pattern recognition receptors (PRRs) mainly include retinoic acid-inducible gene I (RIG-I)-like receptors (RLRs), Toll-like receptors (TLRs), cytosolic DNA sensors, and nucleotide-binding oligomerization domain (NOD)-like receptors (NLRs), whose activation is the first line of defense for initiating the innate immune response [97]. Viral DNA or RNA is the major pathogen-associated molecular pattern (PAMP) that activates various PRRs [98]. RLRs include RIG-I and melanoma differentiation-associated gene 5 (MDA5), which initiate anti-RNA virus responses by recruiting mitochondrial antiviral-signaling protein (MAVS) [99]. Meanwhile, the cyclic GMP-AMP (cGAMP) synthase (cGAS) responds to DNA virus stimulation by recruiting the stimulator of interferon genes (STING) protein [100]. Next, activated MAVS and STING further recruit and activate tumor necrosis factor receptor-associated factor (TRAF) signaling, promoting type I and proinflammatory cytokines by activating the downstream key transcription factors IRF3, IRF7, and NF-κB. IFN and proinflammatory cytokine synthesis induce an anti-DNA/RNA virus response [101].

### 4.1 Role of OTUDs Proteins in Innate Anti-RNA Virus Responses

**Figure 8:**
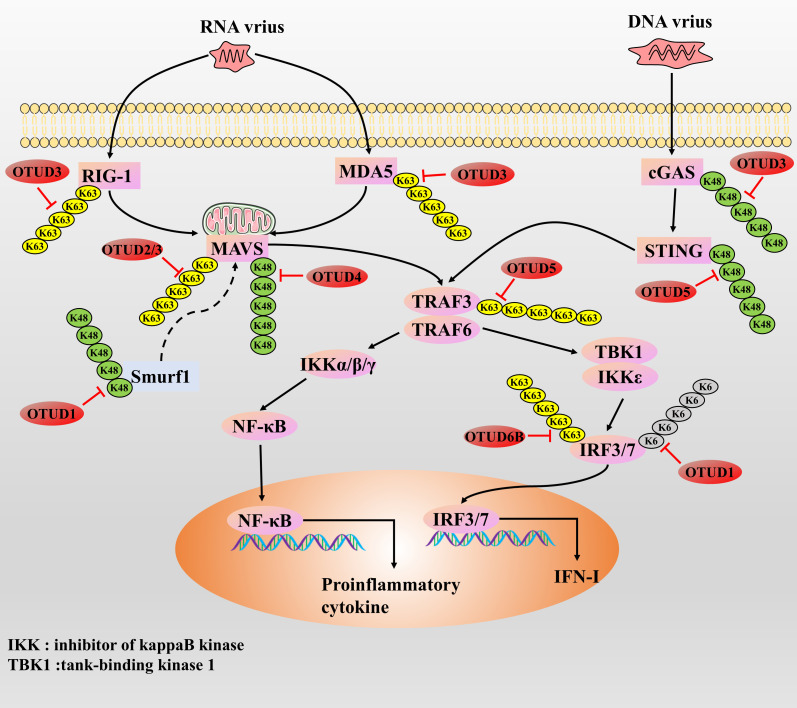
The role of OTUDs proteins in the antiviral response

Several studies have shown that OTUD proteins are mainly involved in the regulatory process of suppressing RNA virus-induced innate immune responses. RNA viruses have been reported to promote the expression of DNA damage-inducible transcript 3, which supports the expression of OTUD1 through activation of the NF-κB axis. Then OTUD1 further inhibits RNA virus-induced type I interferon (IFN-I) production but does not affect DNA-STING signaling, which may be a possible host mechanism for preventing an overly anti-RNA viral response as a mechanism for the host to avoid excessive anti-RNA virus responses [102]. They demonstrated that OTUD1 promotes the accumulation of Smurf1 protein by removing the K48-type polyUb chain of Smurf1 (an E3 ubiquitin ligase), which then induces the degradation of MAVS protein through ubiquitination, suggesting that OTUD1 indirectly inhibits the MAVS/TRAF3/TRAF6 axis in a Smurf1-dependent manner to regulate the anti-RNA virus responses negatively [101]. Alternatively, OTUD1 can directly cleave the K6-type polyUb chain of the IRF3 protein, leading to diminished binding of IRF3 to the promoter and thus negatively regulating the anti-RNA virus response [103]. In contrast, OTUD1 deficiency leads to RNA/RLR signaling activation, which promotes the synthesis of IFN-I and proinflammatory cytokines and enhances the host’s ability to mount an anti-RNA virus response [103]. However, mutations in OTUD1 function over-enhance the immune response, leading to the development of autoimmune diseases [104]. Thus, OTUD1 plays an important role in maintaining immune homeostasis. Similar to the function of OTUD1, OTUD2 is also upregulated during RNA virus infection and can inhibit anti-RNA virus responses and promote viral replication by inhibiting the aggregation of MAVS proteins through direct targeted cleavage of the K63-type polyUb chain [39]. In the uninfected state, OTUD3 can be activated by acetylation modification; however, in the infected state, the virus removes the deacetylation modification of OTUD3 by recruiting the deacetylase SIRT1, which inhibits the deubiquitinating enzyme activity of OTUD3 [52,97]. It was found that OTUD3 can inhibit anti-RNA virus responses by modifying two types of targets. First, OTUD3 can directly cleave the K63-type polyUb chain of the MAVS protein, blocking MAVS aggregation and activation [52]. Alternatively, OTUD3 is also able to remove the K63-type poly-Ub chains attached to RIG-1 and MDA5 proteins, thereby indirectly blocking the aggregation and activation of MAVS by inhibiting the activities of RIG-1 and MDA5 proteins, and ultimately inhibiting the anti-RNA virus response [97]. However, since the acetylation level of OTUD3 is reduced upon viral infection, the RLR/MAVS axis will undergo partial activation in response to RNA viruses and initiate an anti-RNA virus response [52]. Then, the authors also further analyzed the correlation between the acetylation level of OTUD3 and the expression level of IFN-β in the serum of influenza patients, and they found that the acetylation level of OTUD3 showed a significant negative correlation with the expression level of IFN-β [52]. Currently, the study of OTUD4 and antiviral response is somewhat controversial. In the face of RNA virus infection such as Sendai virus (SeV) or vesicular stomatitis virus (VSV), OTUD4-dependent upregulation of the transcriptional activity of IRF3/7 occurs and inhibits its degradation by cleaving the K48-type poly-Ub chain of the MAVS protein, which facilitates innate antiretroviral signaling, and the knockdown of OTUD4 significantly inhibited RNA virus-triggered signaling [105]. Conversely, recent studies have confirmed that OTUD4 inhibits innate anti-RNA virus signaling by blocking Zika virus replication by inhibiting the p38 MAPK signaling pathway [106]. Thus, the mechanism by which OTUD4 regulates innate anti-RNA virus responses needs further investigation to clarify the possibility of both outcomes. In response to the Sendai virus, OTUD5 inhibits the anti-RNA virus response by blocking the downstream IFN-I signaling response through cleavage of the K63-type polyUb chain of the TRAF3 protein [107]. In addition, overexpression of OTUD6B inhibited the cellular antiviral response, whereas OTUD6B knockdown enhanced antiviral resistance and survival in carp and grass carp [108]. The authors found that OTUD6B was upregulated during viral infection, and it inhibited the activity of IRF3/7 proteins by cleaving the K63-type polyUb chain and inhibiting the phosphorylation process, exerting an inhibitory anti-RNA virus effect [108] (Fig. 8).

RNA vrius recruits MAVS by actives the RIG-I and MDA5. cGAS responds to DNA virus stimulation by recruiting the STING protein. Next, activated MAVS and STING further recruit and activate TRAF protein, promoting type I and proinflammatory cytokines by activating the downstream key transcription factors IRF3, IRF7, and NF-κB. IFN and proinflammatory cytokine synthesis induce an anti-DNA/RNA virus response.

### 4.2 Role of OTUDs Proteins in Innate Anti-DNA Viral Responses

Currently, there are fewer studies on OTUD proteins and innate anti-DNA viral responses, with only OTUD3 and OTUD5. Whether enhancing or inhibiting, most enzymes that regulate the antiviral response usually respond to DNA and RNA viral infections in the same way, showing little to no opposing effects. Earlier, we mentioned that OTUD3 can inhibit the anti-RNA virus response. In addition, OTUD3 is also able to inhibit cGAS degradation by removing the K48-type polyUb chain to which the cGAS protein is attached, thereby initiating the anti-DNA virus response [97]. However, since the acetylation level of OTUD3 is reduced upon viral infection, the cGAS/STING axis will respond to partially blocked DNA viruses to prevent excessive anti-DNA viral responses [52]. As with OTUD3, in studies of herpes simplex virus type I and OTUD5, it was demonstrated that OTUD5 promotes DNA/cGAS/STING/IRF3 signaling through cleavage of the K48-type poly-Ub chain of the STING protein to enhance the anti-DNA viral response of the host [109] (Fig. 8). In summary, OTUD3 and OTUD5 regulate anti-RNA and anti-DNA virus responses in opposite ways, suggesting that OTUD3 and OTUD5 have some complexity in regulating host antiviral immune responses, which needs to be investigated in greater depth.

## 5 Summary and Outlook

In recent years, DUBs have gradually emerged as emerging targets in therapeutic studies of cancer and immune diseases. In this review, we comprehensively summarize the roles of seven DUBs with OTU structural domains in cancer progression and antiviral immune responses, including OTUD1, OTUD2, OTUD3, OTUD4, OTUD5, OTUD6A, and OTUD6B. OTUD proteins affect the degradation of substrate proteins and cellular transport processes by cleaving different types of Ub chains. It affects tumor cells’ signal transduction, cell proliferation, invasion, and drug-resistance behaviors. Interestingly, we found that OTUD proteins play opposing roles in different tissue types of human cancers. Since different cancer tissue types may have widely differing tumor microenvironments, the same OTUD proteins may play different cancer regulatory roles with tissue specificity, which may explain the occurrence of this phenomenon. Therefore, each cancer should examine such genes to determine whether they play a tumor-suppressive or tumor-promoting role. Currently, studies of corresponding OTUDs targeting inhibitors or activators are lacking, and more *in vivo* and *in vitro* studies on OTUDs may help develop inhibitors or agonists targeting OTUDs. It is worth pondering that due to the dual action, cancer therapies targeting OTUDs, although they may be effective in some tissue cancers, may induce the risk of cancerous transformation in other tissues, which requires more studies for in-depth analysis. Therefore, more in-depth studies are still needed to explore the role of OTUD proteins in different cancers and the related regulatory mechanisms. In the future, such scientific studies should be carried out gradually. In addition, we also summarized the link between OTUDs and host antiviral response. We found that OTUDs can inhibit the RNA virus-induced innate immune response process to prevent the development of autoimmune diseases, while the role of OTUD4 is still controversial. In contrast, only OTUD3 and OTUD5 have been found to enhance the host’s anti-DNA virus response. These studies suggest that OTUDs are deeply involved in developing antiviral immune responses and emphasize their importance as targets for drug therapy. On the other hand, targeted inhibition of OTUD protein may suppress the host’s antiviral response, leading to a decrease in host immune capacity; On the contrary, targeting and promoting OTUD protein may lead to the occurrence of autoimmune diseases. Therefore, when conducting anti-cancer research, attention should be paid to changes in host immune function. In conclusion, based on the current relevant studies, OTUBs may become an emerging, potential, and reliable target gene or protein, which still requires further studies to illustrate the important role of OTUBs in regulating cancer progression and antiviral response.

## Data Availability

Not applicable.
